# RETRACTED: Zhang et al. MicroRNA-378 Alleviates Cerebral Ischemic Injury by Negatively Regulating Apoptosis Executioner Caspase-3. *Int. J. Mol. Sci.* 2016, *17*, 1427

**DOI:** 10.3390/ijms262311476

**Published:** 2025-11-27

**Authors:** Nan Zhang, Jie Zhong, Song Han, Yun Li, Yanling Yin, Junfa Li

**Affiliations:** 1 Department of Human Anatomy, School of Basic Medical Sciences, Capital Medical University, Beijing 100069, China; nanzhang@mail.ccmu.edu.cn; 2 Department of Neurobiology and Center of Stroke, Beijing Institute for Brain Disorders, Capital Medical University, Beijing 100069, China; zhongjie198793@sina.com (J.Z.); songhan@ccmu.edu.cn (S.H.); yun_li@ccmu.edu.cn (Y.L.); yyling@ccmu.edu.cn (Y.Y.)

The journal retracts the article “MicroRNA-378 Alleviates Cerebral Ischemic Injury by Negatively Regulating Apoptosis Executioner Caspase-3” [1] cited above.

Following publication, concerns were raised by the Editorial Office regarding the integrity of the images presented in this article [1].

Adhering to our standard procedure, an investigation was conducted by the Editorial Office and Editorial Board that identified indications of editing and partial duplication in Figure 2 as well as apparent errors in the bar graphs of Figure 4. Despite the authors’ cooperation in this process, the full raw material required to meet the journal’s requirements for original images could not be provided for Editorial Board evaluation. Consequently, the Editorial Board has lost confidence in the reliability of the findings and has decided to retract this publication, as per MDPI’s retraction policy (https://www.mdpi.com/ethics#_bookmark30).

This retraction was approved by the Editor-in-Chief of the *International Journal of Molecular Sciences*.

The authors did not provide a comment on this decision.
